# Proinflammatory Cytokines and Potassium Channels in the Kidney

**DOI:** 10.1155/2015/362768

**Published:** 2015-10-05

**Authors:** Kazuyoshi Nakamura, Hikaru Hayashi, Manabu Kubokawa

**Affiliations:** Department of Physiology, Iwate Medical University School of Medicine, 2-1-1 Nishitokuta, Yahaba, Iwate 028-3694, Japan

## Abstract

Proinflammatory cytokines affect several cell functions via receptor-mediated processes. In the kidney, functions of transporters and ion channels along the nephron are also affected by some cytokines. Among these, alteration of activity of potassium ion (K^+^) channels induces changes in transepithelial transport of solutes and water in the kidney, since K^+^ channels in tubule cells are indispensable for formation of membrane potential which serves as a driving force for the transepithelial transport. Altered K^+^ channel activity may be involved in renal cell dysfunction during inflammation. Although little information was available regarding the effects of proinflammatory cytokines on renal K^+^ channels, reports have emerged during the last decade. In human proximal tubule cells, interferon-*γ* showed a time-dependent biphasic effect on a 40 pS K^+^ channel, that is, delayed suppression and acute stimulation, and interleukin-1*β* acutely suppressed the channel activity. Transforming growth factor-*β*1 activated KCa3.1 K^+^ channel in immortalized human proximal tubule cells, which would be involved in the pathogenesis of renal fibrosis. This review discusses the effects of proinflammatory cytokines on renal K^+^ channels and the causal relationship between the cytokine-induced changes in K^+^ channel activity and renal dysfunction.

## 1. Introduction

Renal tubular potassium (K^+^) channels are involved in a wide spectrum of the transepithelial transport in the kidney [1, 2]. They contribute to the formation of the cell-negative potential, which serves as a driving force for the electrogenic passive transport of solutes, such as apical Na^+^ entries through the Na^+^-glucose cotransporter (SGLT) in proximal tubule cells and the epithelial Na^+^ channel (ENaC) in the principal cells of cortical collecting duct (CCD) [1, 2]. The apical K^+^ channels in the principal cells of CCD are the major pathway of K^+^ secretion [1, 2]. The renal tubular K^+^ channels also play important roles in K^+^ recycling for the apical Na^+^-K^+^-2Cl^−^ cotransporter (NKCC) in the thick ascending limb (TAL) and the basolateral Na^+^-K^+^ ATPase along the nephron [1, 2]. In addition to the physiological importance, the renal tubular K^+^ channels seem to be involved in the pathogenesis of renal cell injury or renal dysfunction. Some investigators reported that the blockade of K^+^ channel activity ameliorated hypoxic renal cell injury [3–5]. Others reported that the decrease in K^+^ channel activity exacerbated renal ischemia/reperfusion injury [6, 7] or endotoxemic renal failure [8].

The cytokine family comprises a variety of multifunctional proteins, which play pivotal roles in immune modulation and inflammation [9, 10]. Almost all organs are subjected to the modulatory effects of cytokines. The nervous system [11, 12], cardiovascular system [13], respiratory system [14], gastrointestinal tracts [15], and kidney [16] are also targets of cytokines. It is widely accepted that cytokines are secreted mainly by immune cells in response to microbial infection. However, other cell types are also capable of secreting cytokines [9, 10]. The kidney proximal tubule cells produce proinflammatory cytokines in response to lipopolysaccharide (LPS) [17] or albumin [18]. It is thought that the proximal tubule cells function as the proinflammatory cells or immune responders, which play roles in the pathogenesis of renal dysfunction [19, 20]. The TAL [21, 22] and collecting ducts [23] are also the sources of proinflammatory cytokines. Furthermore, renal tubule cells express specific receptors which mediate effects of individual cytokines [24–26].

Several proinflammatory cytokines, such as interferon-*γ* (IFN-*γ*), interleukin-1*β* (IL-1*β*), and tumor necrosis factor-*α* (TNF-*α*), have been reported to affect Na^+^ reabsorption in renal tubular epithelia [27–34]. Considering that the driving force of the transepithelial Na^+^ reabsorption is dependent on the K^+^ channel activity, as well as Na^+^-K^+^-ATPase [1, 2], it is important to know whether cytokines would affect the K^+^ channels. These proinflammatory cytokines were also reported to cause cell injury in various organs, including the kidney. Since the alterations in K^+^ channel activity were involved in renal cell injury as described above, it is possible that the cytotoxic effects of proinflammatory cytokines would partly be mediated by their action on renal K^+^ channels. To date, however, information is restricted, regarding the effects of cytokines on K^+^ channel activity in renal tubule epithelia. This is in sharp contrast to the accumulating evidence for the effects of cytokines on neuronal ion channels [35]. In this review, we discuss the effects of proinflammatory cytokines on renal tubular K^+^ channels and the relevance of such effects to renal cell damage.

## 2. Effects of Cytokines on K^**+**^ Channels in the Proximal Tubule

In the kidney, the proximal tubule reabsorbs about 70% of Na^+^ filtered in glomeruli [1, 2]. The basolateral K^+^ channels provide the driving force for the apical Na^+^ entry by forming the cell-negative potential and serving as the K^+^ recycling pathway coupled with the Na^+^-K^+^-ATPase activity [1, 2]. The effects of cytokines on the K^+^ channels in human proximal tubule cells have recently been reported, using the patch-clamp technique [36–38]. In cultured human proximal tubule cells (RPTECs) derived from the normal kidney, an inwardly rectifying K^+^ channel with an inward conductance of 40 pS is predominantly observed under the control condition [39]. Although the molecular characteristic of this K^+^ channel is still unknown, electrophysiological studies revealed various functional properties. The 40 pS K^+^ channel possesses a relatively high open probability (0.8 on average) with no voltage dependence [39] and contributes to the formation of cell-negative potential [40]. Furthermore, the activity of this K^+^ channel is regulated by intracellular ATP [39], pH [39], and protein phosphorylation processes mediated by protein kinase A (PKA) [39] and protein kinase C (PKC) [38, 41], all of which are consistent with the properties of the basolateral K^+^ channels in animal proximal tubule cells [1, 2, 42].

**Figure 1:**
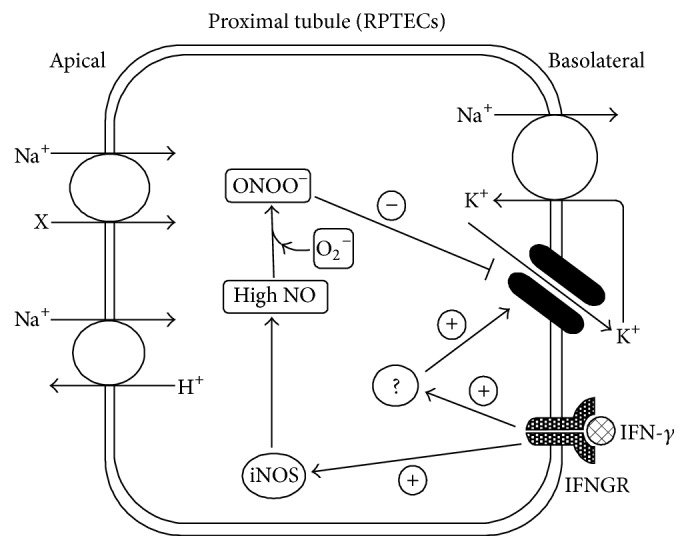
Illustration of the time-dependent biphasic effect of IFN-*γ* on the 40 pS K^+^ channel in RPTECs. Prolonged treatment of cells with IFN-*γ* greatly enhances iNOS expression, which results in generation of a large amount of NO. The excessive NO reacts with superoxide (O_2_^−^) to form peroxynitrite (ONOO^−^), which impairs the 40 pS K^+^ channel by oxidation and/or nitrosylation. IFN-*γ* also acutely activates the 40 pS K^+^ channel by unknown mechanisms. IFNGR: IFN-*γ* receptor.

It was reported that two proinflammatory cytokines, IFN-*γ* [36, 37] and IL-1*β* [36, 38], affected the activity of the 40 pS K^+^ channel in RPTECs. IFN-*γ* possessed a time-dependent biphasic effect on the 40 pS K^+^ channel: a delayed suppressive effect and an acute stimulatory one [36, 37]. Both effects were blocked by inhibitors of Janus kinase (JAK) which was closely related to the IFN-*γ* receptor, suggesting that the effects of IFN-*γ* would be receptor specific [37]. In fact, human proximal tubule cells were reported to express IFN-*γ* receptors [24]. The delayed suppressive effect of IFN-*γ* on channel activity was mediated, at least in part, by overproduction of NO in RPTECs [36, 37]. It was reported that NO stimulated activity of the 40 pS K^+^ channel in RPTECs at low concentrations (micromolar level) through activation of the cGMP/protein kinase G (PKG) pathway, whereas it suppressed channel activity at higher concentrations (millimolar level) [40]. The mechanism for the delayed suppressive effect of IFN-*γ* is depicted in Figure 1. When RPTECs were treated with IFN-*γ* for 24 h, expression of inducible NO synthase (iNOS) was greatly enhanced, producing a large amount of NO. The excessive NO reacted with superoxide and generated peroxynitrite [37]. This peroxynitrite would suppress the K^+^ channel activity by oxidating or nitrosylating the channel and/or its related proteins [37, 40]. Thus, the responses of the channels to NO modulators were reversed in IFN-*γ*-treated cells, compared to the control cells [36, 37]. A NOS inhibitor, L-NAME, stimulated channel activity and a NO donor, L-arginine, suppressed channel activity in IFN-*γ*-treated cells, whereas L-NAME suppressed channel activity and L-arginine stimulated channel activity in control cells [36, 37]. With regard to the acute stimulatory effect of IFN-*γ*, the mechanisms involved are currently obscure (Figure 1). Although the activity of the 40 pS K^+^ channel was upregulated by PKA- and PKG-mediated phosphorylation processes [39, 43], inhibitors of these protein kinases failed to block the stimulatory effect of IFN-*γ* [37]. Phosphatidylinositol-3-kinase (PI3 K) is one of the molecules that mediate the IFN-*γ* signaling [44]. However, PI3 K inhibitors were also ineffective in blocking the IFN-*γ*-induced activation of the channel [37].

**Figure 2:**
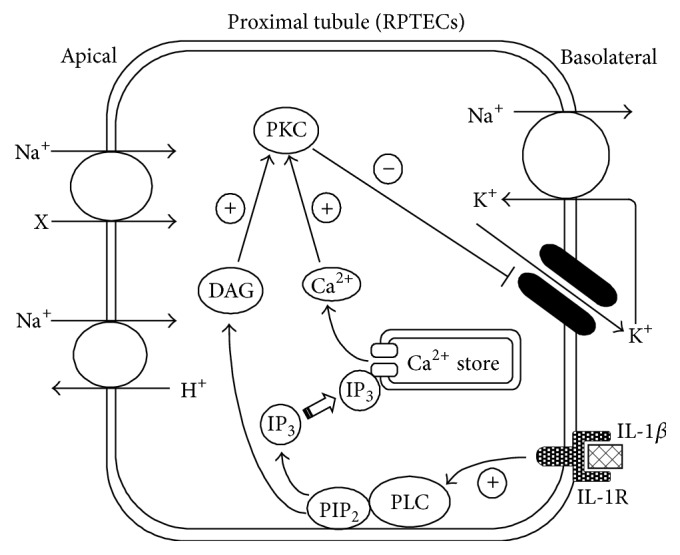
Illustration of the acute effect of IL-1*β* on the 40 pS K^+^ channel in RPTECs. IL-1*β* activates phospholipase C (PLC), which generates diacylglycerol (DAG) and 1,4,5-trisphosphate (IP_3_) from phosphatidylinositol 4,5-bisphosphate (PIP_2_) in the cytoplasmic membrane. Binding of IP_3_ to the IP_3_ receptor of the intracellular Ca^2+^ sore releases Ca^2+^. Both the DAG and increased intracellular Ca^2+^ activate PKC, which consequently phosphorylates the channel and/or its related proteins, suppressing the channel activity. IL-1R: IL-1 receptor.

IL-1*β* also acutely affected the activity of the 40 pS K^+^ channel in RPTECs and the mode of action was suppressive [36, 38]. The effect of IL-1*β* was highly likely receptor mediated, since the IL-1 receptor antagonist (IL-1RA) completely abolished it [38]. With regard to the existence of IL-1 receptors in the kidney, it was reported that the type I IL-1 receptors were widely distributed in renal tubules, as well as glomeruli [25]. In addition to IL-1RA, the acute suppressive effect of IL-1*β* was blocked by a PKC inhibitor and an inhibitor of phospholipase C [38]. These observations strongly suggested that the effect of IL-1*β* was dependent on the PKC pathway (Figure 2). In support of this notion, PKC directly suppressed the activity of the 40 pS K^+^ channel in inside-out patches [38], and fluorescent Ca^2+^ imaging using Fura 2 revealed that IL-1*β* increased intracellular Ca^2+^ [38], which was prerequisite for the activation of conventional PKC [45]. Furthermore, the PKC-dependent effects of IL-1*β* were also observed in other ion channels, including the hippocampal Ca^2+^ channel [46], the middle ear Na^+^ channel [47], the intestinal Cl^−^ channel [48], and the K^+^ channel in the cultured mouse CCD cell line, M1 cells (our unpublished observation). Although several investigators reported that IL-1*β* increased iNOS expression in some tissues, such effect was not observed in RPTECs [36] or the nonpassaged primary culture of human proximal tubule cells [49]. Thus, IL-1*β* did not possess the NO-dependent delayed suppressive effect on the 40 pS K^+^ channel, which was demonstrated by IFN-*γ*.

Huang et al. [50] have recently reported that transforming growth factor-*β*1 (TGF-*β*1) upregulated the KCa3.1 channel in immortalized human proximal tubule cells, HK2. According to their report, the whole-cell current sensitive to TRAM34, an inhibitor of KCa3.1, was profoundly increased after treatment of HK2 cells with TGF-*β*1 for 48 h, while the TRAM34-sensitive current was not observed in control cells. The mRNA expression of KCa3.1 was also increased by TGF-*β*1. KCa3.1 is an intermediate conductance Ca^2+^-activated K^+^ channel and widely distributed throughout the body, excepting most of excitable tissues [51]. Much more attention has been paid to this Ca^2+^-activated K^+^ channel because of its pathological relevance in various diseases, including asthma, atherosclerosis, autoimmunity, and renal fibrosis [51]. Indeed, Huang et al. [50] suggested that the activation of KCa3.1 would play an important role in the TGF-*β*-induced renal fibrosis. TGF-*β* increased KCa3.1 activity, which in turn contributed to the activation of mitogen-activated protein kinase signaling and increased expression of monocyte chemoattractant protein-1 [50].

## 3. Effects of Cytokines on K^**+**^ Channels in the TAL and CCD

**Figure 3:**
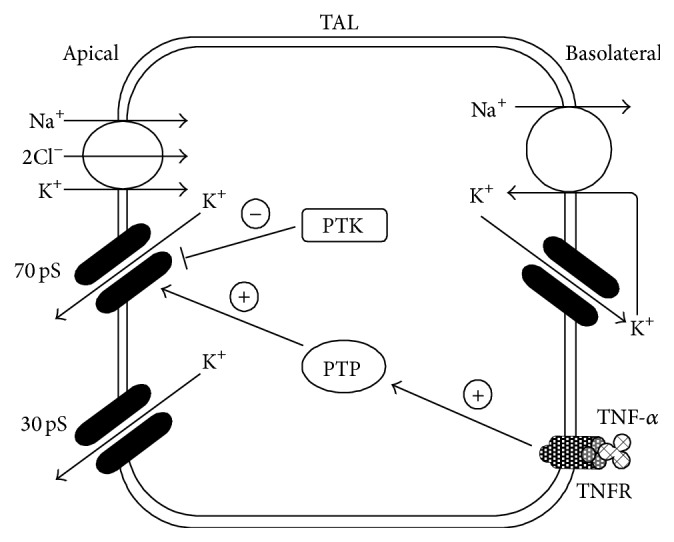
Illustration of the acute effect of TNF-*α* on the 70 pS K^+^ channel in the TAL. TNF-*α* stimulates PTP activity, which causes tyrosine dephosphorylation of the 70 pS K^+^ channel and activates it. TNFR: TNF receptor.

The first report that cytokines affect K^+^ channel activity was presented in 2003 by Wei et al. [52], who clearly demonstrated that TNF acutely stimulated activity of a 70 pS K^+^ channel in the apical membrane of rat TAL, using the patch-clamp technique. Although the PKA- and nitric oxide- (NO-) dependent pathways had been shown to stimulate the activity of the 70 pS K^+^ channel [53, 54], both a PKA inhibitor and an inhibitor of NO synthase did not affect the TNF-induced activation of the channel [52]. In contrast, an inhibitor of protein tyrosine phosphatase (PTP) blocked the stimulatory effect of TNF on channel activity [52]. Furthermore, an inhibitor of protein tyrosine kinase (PTK) increased channel activity, and the subsequent administration of TNF in the presence of the PTK inhibitor did not cause additional increase in channel activity [52]. It was also demonstrated that TNF significantly increased the PTP activity in cultured rat TAL cells [52]. Therefore, they concluded that the stimulatory effect of TNF on the 70 pS K^+^ channel was dependent on tyrosine dephosphorylation processes mediated by PTP [52], as shown in Figure 3.

It is well known that a ROMK-like 30 pS K^+^ channel, as well as the 70 pS K^+^ channel, is present in the apical membrane of TAL [52, 53]. Both K^+^ channels contribute to the K^+^ recycling across the apical membrane of the TAL, which maintains the normal function of the NKCC [1, 2]. Wei et al. [52] also found that TNF did not affect the activity of this 30 pS K^+^ channel.

With regard to ROMK, there are also reports demonstrating that the gene expression of this K^+^ channel was modulated by cytokines [32]. Schmidt et al. [32] reported that administration of proinflammatory cytokines, such as IL-1*β*, IFN-*γ*, and TNF-*α*, to the mouse decreased mRNA expression of ROMK in the whole kidney. Although the systemic administration of cytokines resulted in hypotension and reduced blood flow in the kidney, renal ischemia itself had no apparent effect on ROMK gene expression [32]. In addition, IL-1*β*, IFN-*γ*, and TNF-*α* suppressed ROMK gene expression in the mouse CCD cell line to the same extent observed in the whole animal experiments [32]. Therefore, they strongly suggested that the proinflammatory cytokines directly suppressed the ROMK gene expression [32].

## 4. Cytokine-Induced Changes in Activities of K^**+**^ Channels and Transporters in the Kidney

The activities of renal tubular K^+^ channels are functionally coupled to various transporters [1, 2]. Basolateral K^+^ channels in proximal tubule cells cooperate with the basolateral Na^+^-K^+^ ATPase to provide a driving force for Na^+^ entry through the apical transporters, such as SGLT [1, 2]. Similar cooperation of basolateral K^+^ channels with the Na^+^-K^+^ ATPase also exists in distal nephron segments [1, 2]. In the TAL, apical K^+^ channels serve as the K^+^ recycling pathways for the apical NKCC, which facilitates reabsorption of Na^+^ and Cl^−^ [1, 2]. In principal cells of CCD, apical Na^+^ entry through the ENaC is partly dependent on the cell-negative potential generated by apical and basolateral K^+^ channels [1, 2]. Thus, the changes in renal K^+^ channel activity would result in alterations in the transport activity of solutes and water. If the transport activity in the kidney drastically changes, the homeostasis of body fluid would be profoundly perturbed.

In contrast to the renal K^+^ channels, there are relatively many reports demonstrating the effects of cytokines on renal transporters. TNF-*α* reduced ouabain-sensitive ^86^Rb^+^ uptake in the TAL [21] and NKCC expression in the kidney [31, 33]. These observations are inconsistent with the stimulatory effect of TNF-*α* on the 70 pS K^+^ channel in the TAL reported by Wei et al. [52]. The apical 70 pS K^+^ channel acts as the recycling pathway for the apical NKCC [1, 2]. Therefore, the TNF-induced increase in channel activity should be related to increased Na^+^ transport. Such a discrepancy would partly be accounted for by the temporal factor. TNF-*α* reduced ^86^Rb^+^ uptake after 24 h [21] and NKCC expression after 4 h [31] or 7 days [33], whereas stimulation of channel activity by TNF-*α* occurred in a few minutes [52]. Besides the acute stimulatory effect on K^+^ channel, TNF-*α* induces expression of cyclooxygenase (COX) which subsequently generates prostaglandin E_2_ (PGE_2_) [55]. It was reported that PGE_2_ suppressed the activity of the 70 pS K^+^ channel in the TAL [56]. The effects of TNF-*α* on channel activity might be time-dependently biphasic similar to the effect of IFN-*γ* on K^+^ channel activity in RPTECs. If so, the delayed COX/PGE_2_-dependent channel suppression would be consistent with the TNF-*α*-induced inhibition of transport activity and transporter expression. TNF-*α* was also reported to increase SGLT2 expression in the porcine proximal tubule cell line, LLC-PK_1_ [57], which well fits with the stimulatory effect of TNF-*α* on K^+^ channel activity. IFN-*γ* suppresses gene expression of various transporters. These include Na^+^-K^+^ ATPase [32, 58, 59], Na^+^-H^+^ exchanger (NHE) [32], Na^+^-Ca^2+^ exchanger [60], Na^+^-Cl^−^ cotransporter (NCC) [58], NKCC [32], SGLT [59], glucose transporters [59], and urea transporters [61]. On the other hand, IFN-*γ* seems to be involved in the upregulations of NKCC and NCC in the distal nephron and NHE in the proximal tubule, which would contribute to the salt and water retention during the angiotensin II-induced hypertension [34]. The stimulatory effect of IFN-*γ* on K^+^ channel activity might play a role in such an upregulated Na^+^ transport. Similar to TNF-*α* and IFN-*γ*, the effects of IL1-*β* on renal transporters are mainly suppressive, which is in good agreement with its suppressive effect on K^+^ channel activity. IL-1*β* inhibits Na^+^ reabsorption in the CCD [27] and expression of Na^+^-K^+^ ATPase in medullary and cortical kidney cells [28] or cultured LLC-PK_1_ cells [29]. It also reduces many other transporters, as was observed with IFN-*γ* [32, 58, 59, 61].

## 5. Effects of Cytokines on K^**+**^ Channel Activity and Renal Cell Injury

The effects of proinflammatory cytokines on renal K^+^ channels would be implicated in renal dysfunction or renal cell injury. Proinflammatory cytokines are the key molecules, which promote cell injury in many organs during inflammatory diseases [9, 10]. They are involved in the pathogenesis of acute kidney injury and chronic kidney disease [62, 63]. The endotoxemia-induced acute renal failure is one of the clinical manifestations that highlight the detrimental effects of proinflammatory cytokines on the kidney [64]. Therapeutic use of cytokines sometimes brings about adverse effects. For example, application of IFNs in renal cell carcinoma or viral hepatitis may well result in undesirable severe renal dysfunction [65]. Furthermore, it has been reported that proinflammatory cytokines cause glomerulonephritis accompanied by proteinuria, tubulointerstitial fibrosis, and apoptosis/necrosis of tubular cells in various experimental models [66, 67]. These effects are generally thought to be mediated by activation of caspases and various transcription factors [9, 10]. The activated transcription factors initiate synthesis of many effector proteins, such as other cytokines, chemokines, matrix metalloproteases, iNOS, and adhesion molecules [9, 10]. All of these effector proteins could participate in the promotion of renal cell injury. As described below, the changes in K^+^ channel activity themselves could affect cell viability. Thus, it is possible that the cytokine-induced renal cell injury would be mediated by modulation of K^+^ channel activity. In fact, TGF-*β* upregulates a Ca^2+^-activated K^+^ channel in HK2 cells, which in turn contributed to the generation of a chemokine crucial for the pathogenesis of renal fibrosis [50]. Modulation of K^+^ channel activity by TNF-*α* was also reported to induce cell death in a rat liver cell line [68] and prolonged action potential duration, which was involved in the sudden cardiac death, in dog cardiomyocytes [69].

Many reports have demonstrated that changes in activity of renal tubular K^+^ channels would be involved in renal cell injury. With regard to the mode of action of K^+^ channel activity on renal cell injury, however, profound controversy exists. An ATP-sensitive K^+^ channel (K_ATP_) blocker, glibenclamide, was reported to reduce hypoxia- or ischemia/reperfusion-induced renal cell injury in isolated rat proximal tubules [3], perfused rat kidneys [4], and rats* in vivo* [5]. Glibenclamide also improved kidney structure and function in the rat model of chronic kidney disease [70]. Blocking the activity of KCa3.1 K^+^ channel by TRAM34 inhibited production of inflammatory mediators, which contributed to renal fibrosis, in HK2 cells [50, 71] or diabetic mice [72]. It was reported that the downregulation of Kir4.1 by intracellular acidification would contribute to cell protection in the rat proximal tubule [73]. These findings suggested that increased K^+^ channel activity would cause renal cell injury. In contrast, other investigators reported that maintaining or increasing K^+^ channel activity is rather protective for renal cells. According to their reports, blockade of K^+^ channel by glibenclamide enhanced renal cell injury in isolated perfused rat kidney [6], LLC-PK_1_ cells [7], HK2 cells [8], and mouse proximal tubule cells [8]. Furthermore, a K_ATP_ opener, nicorandil, was reported to ameliorate ischemia/reperfusion injury in the rat kidney [74, 75]. Another K_ATP_ opener, levosimendan, also protects the kidney against the LPS-induced inflammatory responses [8] and ischemia/reperfusion injury [76]. The discrepant effects of altered K^+^ channel activity among reports would partly be due to the differences in experimental systems and cellular conditions. The precise mechanisms by which changes in K^+^ channel activity cause renal cell injury are not completely revealed. It is likely that the loss of intracellular K^+^ through the activated K^+^ channel facilitates cell shrinkage, which triggers apoptotic volume decrease [77]. Furthermore, changes in K^+^ channel activity alter the driving force for Ca^2+^ entry through Ca^2+^-permeable channels, such as TRP channels [78, 79]. Subsequent changes in intracellular Ca^2+^ concentration will activate or suppress various factors, including apoptotic or inflammatory molecules [50, 71, 72, 78, 79]. It also remained to be elucidated whether the cytotoxic effects of proinflammatory cytokines would actually be mediated by their modulation of renal K^+^ channel activity.

## 6. Conclusion

Renal K^+^ channels play important roles in maintaining the normal transport function of renal tubule epithelia. The kidney sometimes suffers from renal ischemia, endotoxemia, and diabetic nephropathy, where proinflammatory cytokines are produced. However, it was only during the last decade that the effects of proinflammatory cytokines on renal K^+^ channels were reported. The effects of cytokines on K^+^ channels may be involved in alterations of tubular transport or onset of renal cell injury. However, the physiological and pathological significances of proinflammatory cytokines in modulating renal tubular K^+^ channels are not well understood. To complicate the matters, a variety of cytokines with different actions are produced during inflammatory responses. Some cytokines activate renal K^+^ channels, while others suppress the same channels. The complexity of cytokine actions gives rise to difficulties in interpreting the final outcome of their effects. Additional studies are required to further clarify the effects of proinflammatory cytokines on renal K^+^ channels.
